# The impact of *BST1* rs4698412 variant on Parkinson’s disease progression in a longitudinal study

**DOI:** 10.3389/fnagi.2025.1570347

**Published:** 2025-04-16

**Authors:** Hao-Ling Xu, Yu Yang, Li-Na Chen, Yun-Jing Li, Guo-En Cai, Ying-Qing Wang, Yan-Hong Weng, Xiao-Ling Lin, Jing Jian, Xiao-Chun Chen, Qin-Yong Ye

**Affiliations:** ^1^Department of Neurology, Fujian Institute of Geriatrics, Fujian Medical University Union Hospital, Fuzhou, China; ^2^Fujian Key Laboratory of Molecular Neurology, Institute of Clinical Neurology, Institute of Neuroscience, Fujian Medical University, Fuzhou, China; ^3^Department of Neurology, Fuzhou First General Hospital Affiliated with Fujian Medical University, Fuzhou, China

**Keywords:** genetic risk, motor progression, cognition, *BST1* rs4698412, Parkinson’s disease, neurodegeneration

## Abstract

**Background:**

While the *BST1* rs4698412 variant demonstrates a robust association with Parkinson’s disease (PD) susceptibility, its role in modulating PD progression remains unexplored.

**Objectives:**

To evaluate differences in the progression of motor symptoms and cognitive function between PD patients carrying the *BST1* rs4698412 A-allele variant and GG homozygotes.

**Methods:**

Baseline clinical data were collected during their initial visits. Disease severity was assessed using the UPDRS-III scale, while cognitive status was evaluated through the MMSE scale. Follow-up visits were conducted at the same center. Linear mixed-effects models were utilized to compare the rate of changes in motor and cognitive features between the two groups.

**Results:**

A total of 182 PD patients with 74 classified as GG carriers and 108 as GA/AA carriers were enrolled. No significant differences were observed in baseline demographic factors or clinical characteristics. Linear mixed-effects models revealed that GA/AA carriers exhibited a greater rate of change in UPDRS-III score compared with GG carriers (difference of −2.091[0.691] points per year, *P* = 0.003). However, no statistically significant difference in the estimated progression rate of MMSE score was found between the two groups (difference of −0.106 [0.217] points per year, *P* = 0.627).

**Conclusion:**

PD patients carrying the *BST1* rs4698412 A-allelic variant showed more pronounced motor function deterioration than GG carriers, suggesting that *BST1* rs4698412 may serve as a genetic risk factor for disease progression in PD.

## 1 Introduction

Parkinson’s disease (PD) is a chronic, progressive neurological disorder marked by motor symptoms such as bradykinesia, rigidity, resting tremor, and disruption in gait. Non-motor symptoms, encompassing cognitive decline, anosmia, psychological and behavioral irregularities, autonomic dysfunction, and sleep disturbances, may also manifest (Poewe et al., 2017). The pathological characteristics of PD primarily stem from the progressive degeneration of dopaminergic neurons and the accumulation of Lewy bodies in the substantia nigra (Wakabayashi et al., 2013). Numerous studies have explored genetic mutations associated with the occurrence of PD, including *SNCA*, *PINK1*, *DJ-1*, *LRRK2*, *Parkin*, and others. Genome-wide association studies (GWAS) and meta-analyses have identified single nucleotide polymorphisms (SNPs) within the Bone Marrow Stromal Cell Antigen 1 (*BST1*) gene on chromosome 4p15 as new susceptibility loci associated with PD across different races and regions (Guo et al., 2015; International Parkinson Disease Genomics Consortium et al., 2011; Liu et al., 2013; Saad et al., 2011; Satake et al., 2009; Sharma et al., 2012; Simon-Sanchez et al., 2011). Among these variants, the rs4698412 (G → A) allele has garnered particular attention as the subject of extensive study. Accumulating research strongly suggests that both the dominant model (AA + AG vs. GG) and allelic model (A vs. G) of *BST1* rs4698412 demonstrate a significant association with an elevated risk of PD in the Asian population (Chang et al., 2011; Li et al., 2019; Shen et al., 2019; Wang et al., 2015).

To date, several studies have investigated the impact of the allelic variant of *BST1* rs4698412 on clinical presentations. Notably, carriers of the *BST1* rs4698412 GA/AA genotype demonstrated significantly higher Unified Parkinson’s Disease Rating Scale (UPDRS-III) scores (*p* < 0.05) and poorer Timed Up and Go (TUG) test performance compared to GG genotype carriers, indicating more severe motor function and more pronounced gait and balance deficits (Li et al., 2019). Furthermore, a meta-analysis conducted by the COURAGE-PD consortium presented the initial GWAS evidence that the A allele of rs4698412 in the *BST1* gene influences the age at onset (AAO) of PD, resulting in an average earlier AAO of 0.526 years in PD patients (Grover et al., 2022). Additionally, the dominant model of rs4698412 in *BST1* was found to be significantly associated with restless legs syndrome in the Chinese population, a condition that often co-occurs with PD (Huang et al., 2021).

Overall, there was a strong association between the A-allele variant of *BST1* rs4698412 and the susceptibility and severity of clinical features in patients with PD. Nonetheless, the influence of the *BST1* rs4698412 variant on the progression of PD remains unexplored. Consequently, a longitudinal study was conducted to assess differences in motor and cognitive progression between *BST1* rs4698412 A-allele carriers and GG homozygotes among Han Chinese PD patients from southern China.

## 2 Subjects and methods

### 2.1 Ethical compliance

This study was conducted in strict accordance with the ethical principles outlined in the World Medical Association Declaration of Helsinki. The research protocol received formal approval from the Ethics Committee of Fujian Medical University Union Hospital (Approval No. 2023KY178). Written informed consent was obtained from all participants.

### 2.2 Study subjects

A total of 824 primary PD patients were consecutively diagnosed and recruited, adhering to the International Parkinson and Movement Disorder Society (MDS) Clinical Diagnostic Criteria for Parkinson’s disease (MDS-PD Criteria) (Postuma et al., 2015). This recruitment process was conducted by two neurologists from the Neurology Department of Fujian Medical University Union Hospital between 2016 and 2018. Baseline clinical data were collected during their initial visits. Patients subsequently received standard medication and underwent follow-up assessments at the same center. After screening based on inclusion and exclusion criteria, a final cohort of 182 patients was ultimately enrolled in the study. Our study’s inclusion criteria comprised the following: (1) PD patients possessing the *BST1* rs4698412 variant confirmed through genetic tests, (2) well-documented initial clinical and demographic information, (3) completion of baseline assessments and at least one follow-up visit, (4) Hoehn-Yahr (H-Y) Stage ≤ 3, and (5) a demonstrated willingness to participate. Exclusions were applied to patients who (1) exhibited secondary Parkinsonian syndrome or Parkinson-plus syndrome, (2) demonstrated an inability to cooperate with scale evaluations, (3) underwent deep brain stimulation (DBS) during the follow-up period, or (4) presented concomitant disorders such as severe organ dysfunction, endocrine system diseases, hematological diseases, autoimmune diseases, or malignant tumors. The flow chart of participants is provided in Supplementary Figure 1.

### 2.3 Clinical evaluations

Demographic and clinical data were obtained from 182 PD patients, including age, gender, age at onset, ethnicity, educational attainment, disease duration, current medications, comorbidities (hypertension, diabetes), and lifestyle factors (smoking, drinking). The age at onset was defined as the age at which either the patient or their immediate family members first noticed symptoms related to PD. The duration of the disease was the time span between the age at onset and the patient’s initial visit at our hospital. The calculation of the levodopa equivalent daily dose (LEDD) was executed employing the specified conversion formula (Tomlinson et al., 2010). The modified Hoehn-Yahr (H-Y) rating and UPDRS scale, particularly the motor examination component (Part 3, UPDRS-III), were utilized to evaluate the severity of the disease in patients during their off-medication state. Simultaneously, we computed specific scores from the UPDRS-III: resting tremor score (items 20 and 21), rigidity score (item 22), bradykinesia score (items 23–26 and 31), and postural and gait disturbance score (items 27–30). Each of these items is scored on a scale ranging from 0 (indicating the absence of symptoms or normal activity) to 4 (representing the most severe dysfunction or impairment). PD patients were categorized into Akinetic-Rigid (AR), Tremor Dominant (TD), and Mixed (MX) subtypes based on baseline UPDRS score, utilizing Lewis’s method (Lewis et al., 2005) along with Rossi’s modifications (Rossi et al., 2010). For the analysis in our current study, the TD subtype and MX subtype were grouped, following a precedent set by a previous study (Oosterveld et al., 2015). Cognitive assessments were performed using the Mini-Mental State Examination (MMSE) scale.

### 2.4 Genotype detection

MassARRAY technology was utilized to discern the genotype of *BST-1* loci rs4698412 in PD patients. Each participant contributed a peripheral blood sample for genetic analysis. Genomic DNA was extracted from the peripheral blood using established protocols. The design of Polymerase Chain Reaction (PCR) primers and the subsequent single base extension reaction was executed through Sequenom Assay Design 3.1 and synthesized by a biological firm. DNA templates containing the targeted SNP region underwent PCR amplification following the manufacturer’s guidelines. Subsequently, the PCR products underwent shrimp alkaline phosphatase (SAP) purification, followed by a single base extension reaction. The resulting extension products were then deposited onto a Sepectro-CHIP and subjected to analysis using matrix-assisted laser desorption/ionization time-of-flight (MALDI-TOF) mass spectrometry. The genotyping data were subjected to analysis using the Sequenom Mass-ARRAY TYPER software (Sequenom). Both the clinical evaluators and patients were blind to the genotype outcomes.

The participants were categorized into two subgroups: *BST1* rs4698412 GG carriers and GA/AA carriers, stratified according to the genetic test results. These subgroups were subsequently utilized for further comparison and analysis within the context of the present study.

### 2.5 Statistical analysis

For all statistical analyses, assessments were made for data normality and homogeneity of variance using the Shapiro–Wilk test and Levene test, respectively. Variables demonstrating a normal distribution were presented as the mean ± standard deviation (SD), while variables with skewed distribution were expressed as median (M) and interquartile range (IQR). Continuous variables were compared using either Student’s independent samples t-test (for normally distributed data) or the Mann–Whitney U Test (for non-normally distributed data). Gender distribution and the adherence of genotype frequencies to Hardy-Weinberg equilibrium (HWE) were compared using the Chi-squared test.

To investigate the potential association between genotype status and disease severity at baseline, a multivariable linear regression model was constructed. In this model, baseline UPDRS-III score served as the dependent variable, while the independent variables included the genotypes of *BST1* rs4698412 (binary), gender (binary), baseline age, baseline duration of disease, years of education, MMSE score at baseline, LEDD at baseline, comorbidities (hypertension, diabetes), and lifestyle factors (smoking, drinking). Likewise, we employed a multivariable linear regression to explore the relationship between genotype status and baseline MMSE score, with adjustments made for gender (binary), baseline age, baseline duration of disease, UPDRS-III score at baseline, years of education, LEDD at baseline, comorbidities (hypertension, diabetes), and lifestyle factors (smoking, drinking).

Linear mixed-effects models were employed to examine the longitudinal rate of changes in motor score (UPDRS-III) and cognition score (MMSE) between PD patients carrying *BST1* rs4698412 GG genotype and GA/AA genotype. Disease duration served as the time scale, and the models incorporated participant-specific random effects for both random intercepts and random slopes, thereby accounting for correlations in repeated measurements from the same participant. The analysis was adjusted for baseline age, gender (binary), years of education, baseline duration of disease, LEDD at baseline, comorbidities (hypertension, diabetes), and lifestyle factors (smoking, drinking). Furthermore, MMSE score at baseline was included as a covariate in longitudinal motor assessments, while UPDRS-III score at baseline was considered a covariate in longitudinal cognitive evaluations between the two groups.

The analyses were conducted using IBM SPSS Statistics version 26.0 software (SPSS, Chicago, IL, USA). The statistical significance level was set at α = 0.05, and results were considered statistically significant when *P* < 0.05.

## 3 Results

### 3.1 Clinical and demographic features

A total of 182 PD patients who underwent 2 or more times of assessments on the UPDRS-III scale were included in the study, comprising 74 *BST1* rs4698412 GG carriers, 84 GA carriers, and 24 AA carriers. Furthermore, 177 patients (73 GG carriers and 104 GA/AA carriers) among them completed 2 or more times of assessments on the MMSE scale (Supplementary Figures 2, 3).

The genotype frequencies for *BST1* rs4698412 were found to be in accordance with Hardy-Weinberg equilibrium and were genetically representative (χ^2^ = 0, *P* = 1, Supplementary Table 1). The clinical and demographic data of all participants were summarized in Table 1. No significant differences were observed between the GG carriers and the GA/AA carriers in either baseline demographic profiles (age, gender, age at onset, disease duration, years of education, hypertension/diabetes comorbidity, smoking/drinking) or clinical assessments (MMSE score, UPDRS-III total score [resting tremor/rigidity/bradykinesia/postural and gait disturbance subscores], H-Y stage, subtype of PD, or baseline LEDD). Baseline data for the MMSE project were provided in Supplementary Table 2.

**TABLE 1:** Demographic and clinical characteristics of all study subjects.

	GG carriers	GA/AA carriers	*P*-value
Patients, *n*	74	108	NA
Male, *n* (%)	41 (55.4)	54 (50.0)	0.553^a^
Age at baseline, Y	61.0 (54.0, 69.0)	63.5 (55.0, 70.0)	0.203^b^
Age at onset, Y	56.9 ± 9.6	58.3 ± 10.5	0.349^c^
Duration of disease at baseline, y	3.0 (2.0, 6.0)	3.0 (1.0, 5.0)	0.312^b^
Education, y	9.0 (6.0, 12.0)	9.0 (6.0, 12.0)	0.300^b^
LEDD at baseline	375.0 (300.0, 450.0)	325.0 (300.0, 437.5)	0.243^b^
**H-Y baseline stage, *n* (%)**			0.320^a^
1	13 (17.6)	14 (13.0)	
1.5	14 (18.9)	19 (17.6)	
2	12 (16.2)	25 (23.1)	
2.5	29 (39.2)	33 (30.6)	
3	6 (8.1)	17 (15.7)	
UPDRS-III score at baseline	23.5 (16.8, 30.0)	22.5 (17.0, 32.8)	0.971^b^
Resting tremor	2.0 (0.0, 4.0)	2.0 (1.0, 4.8)	0.362^b^
Rigidity	5.0 (2.0, 7.25)	4.0 (2.0, 6.0)	0.308^b^
Bradykinesia	11.0 ± 5.5	11.7 ± 5.6	0.347^c^
Postural and gait disturbance	3.0 (2.0, 4.0)	3.0 (2.0, 4.0)	0.722^b^
MMSE score at baseline	27.0 (24.0, 28.0)	26.5 (23.3, 28.0)	0.889^b^
**Subtype of PD, *n* (%)**			
AR	47 (63.5)	63 (58.3)	0.538^a^
MX + TD	27 (36.5)	45 (41.7)	
Hypertension, *n* (%)	16 (21.6)	22 (20.4)	0.838^a^
Diabetes, *n* (%)	5 (6.8)	7 (6.5)	0.941^a^
Smoking, *n* (%)	7 (9.5)	9 (8.3)	0.792^a^
Drinking, *n* (%)	4 (5.4)	3 (2.8)	0.365^a^

UPDRS, Unified Parkinson’s Disease Rating Scale; H-Y, Hoehn-Yahr stages; y, years; Y, years old; MMSE, Mini-Mental State Examination; LEDD, levodopa equivalent daily dose; NA, not applicable. Variables with normal distribution were represented as mean ± standard deviation, while variables with skewed distribution were expressed as median and interquartile range.

^a^Chi-square test.

^b^Mann–Whitney U test.

^c^Two-independent samples *t*-test.

### 3.2 Cross-sectional study of *BST1* rs4698412 GG and GA/AA carriers

There was no significant difference between the two groups in terms of UPDRS-III scores at baseline after adjusting for gender, baseline age, years of education, baseline duration of disease, MMSE score at baseline, LEDD at baseline, comorbidities (hypertension, diabetes), and lifestyle factors (smoking, drinking). Similar to the motor evaluations, no significant impact of genotype status on baseline MMSE score was detected. The *R*^2^ value for the two regression models were 0.172 (*F* = 3.220, *P* = 0.001) and 0.397 (*F* = 9.872, *P* = 0.000), respectively. The significance of the regression equation was verified. Besides, we found that lower MMSE scores were associated with higher UPDRS-III scores, and vice versa, which suggested that there was a bidirectional influence between the motor and cognitive function of the two groups at baseline (Supplementary Table 3).

### 3.3 Progression analysis in *BST1* rs4698412 GG and GA/AA carriers

**FIGURE 1:**
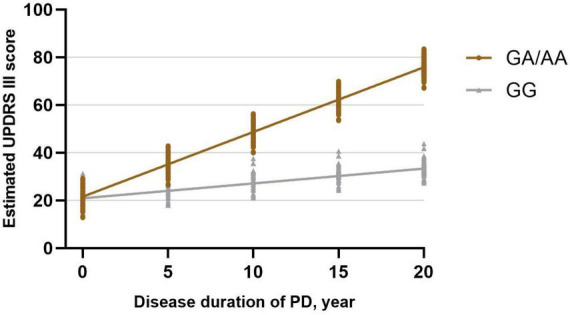
Longitudinal trajectories of UPDRS-III scores in *BST1* rs4698412 GG carriers and GA/AA carriers.

Utilizing a linear mixed-effects model, we further explored the longitudinal rate of variation in UPDRS-III score between GG carriers and GA/AA carriers. Disease duration served as the time scale for this analysis. After adjusting for gender, baseline age, baseline duration of disease, education years, MMSE score at baseline, LEDD at baseline, comorbidities (hypertension, diabetes), and lifestyle factors (smoking, drinking), several significant associations were observed. Specifically, longer duration of disease at baseline (β = 0.879; 95% CI, 0.511 to 1.246; *P* = 0.000) and shorter education period at baseline (β = −0.425; 95% CI, −0.771 to −0.079; *P* = 0.016), and lower MMSE score at baseline (β = −0.446; 95% CI, −0.872 to −0.019; *P* = 0.041) were associated with higher UPDRS III score (Table 2). The estimated rate of progression in the change of UPDRS-III score for GG carriers was 0.622 [0.534] points per year, whereas GA/AA carriers exhibited a higher progression rate of 2.712 [0.439] points per year. Significantly, a notable discrepancy in the rate of UPDRS-III score progression between the two groups was identified (−2.091 [0.691] points per year; *P* = 0.003) (Figure 1).

**TABLE 2:** Models of comparison in rate of change in UPDRS-III score and MMSE score between PD patients with *BST1* rs4698412 GG genotype and GA/AA genotype.

	UPDRS-III score (*n* = 182)		MMSE score (*n* = 177)	
**Characteristics**	**β (95% CI)**	***P*-value**	**β (95% CI)**	***P*-value**
Rate difference	−2.091 (−3.454, −0.727)	**0.003**	−0.106 (−0.534, 0.323)	0.627
Gender (male)	2.740 (−0.145, 5.625)	0.063	0.428 (−0.569, 1.424)	0.398
Age at baseline	0.133 (−0.010, 0.276)	0.068	−0.063 (−0.112, −0.014)	**0.011**
Baseline duration, year	0.879 (0.511, 1.246)	**0.000**	0.025 (−0.081, 0.131)	0.648
Years of education	−0.425 (−0.771, −0.079)	**0.016**	0.458 (0.358, 0.559)	**0.000**
Baseline MMSE score	−0.446 (−0.872, −0.019)	**0.041**	NA	NA
LEDD at baseline	0.005 (−0.003, 0.013)	0.215	0.001 (−0.002, 0.004)	0.427
Baseline UPDRS-III score	NA	NA	−0.064 (−0.108, −0.019)	**0.006**
Hypertension	0.629 (−2.772, 4.030)	0.716	−0.675 (−1.852, 0.503)	0.259
Diabetes	1.868 (−3.377, 7.113)	0.483	0.969 (−0.804, 2.743)	0.282
Smoking	−2.457 (−7.285, 2.371)	0.317	−0.407 (−2.05, 1.234)	0.625
Drinking	3.351 (−3.643, 10.344)	0.346	1.181 (−1.207, 3.569)	0.330

UPDRS, Unified Parkinson’s Disease Rating Scale; MMSE, Mini-Mental State Examination; LEDD, levodopa equivalent daily dose. Bold values indicate statistically significant differences at *p* < 0.05.

When comparing differences in changes within the four subscores of UPDRS-III, the findings indicated that the estimated rate of change in rigidity score among GG carriers (0.309 [0.192] points per year) was lower than that among GA/AA carriers (1.073 [0.158] points per year; difference, −0.764 [0.249] points per year; *P* = 0.002). Besides, the estimated rate of change in bradykinesia score was higher in GA/AA carriers (1.043[0.247] points per year) compared with GG carriers (0.230 [0.302] points per year; difference, −0.813 [0.390] points per year; *P* = 0.038). However, the rates of change in rest tremor score (*P* = 0.185) and postural and gait disturbance score (*P* = 0.052) did not exhibit significant differences between the two groups (Table 3).

**TABLE 3:** Models of comparison in rate of change in UPDRS-III subscore between PD patients with *BST1* rs4698412 GG genotype and GA/AA genotype^a^.

Outcome	β (95% CI)	*P*-value
Resting tremor subscore	−0.236 (−0.586, 0.114)	0.185
Rigidity subscore	−0.764 (−1.254, −0.274)	**0.002**
Bradykinesia subscore	−0.813 (−1.581, −0.045)	**0.038**
Postural and gait disturbance subscore	−0.283 (−0.568, 0.003)	0.052

UPDRS, Unified Parkinson’s Disease Rating Scale. ^a^Covariates including gender, baseline age, duration of disease at baseline, years of education, MMSE score at baseline, LEDD at baseline, comorbidities (hypertension, diabetes), and lifestyle factors (smoking, drinking) were adjusted in each model. Bold values indicate statistically significant differences at *p* < 0.05.

**FIGURE 2:**
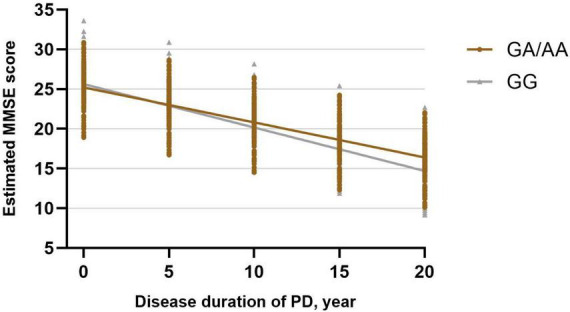
Longitudinal trajectories of MMSE total scores in *BST1* rs4698412 GG carriers and GA/AA carriers.

Subsequently, a linear mixed-effects model was employed to analyze the longitudinal rate of change in the MMSE score between GG carriers and GA/AA carriers, using a similar approach. The analysis was adjusted for baseline age, gender, baseline duration of disease, education years, UPDRS-III score at baseline, LEDD at baseline, comorbidities (hypertension, diabetes), and lifestyle factors (smoking, drinking). The estimated progression rate of change in MMSE score for GG carriers and GA/AA carriers was −0.547 [0.167] points per year and −0.441 [0.139] points per year, respectively. Notably, there was no statistically significant difference in the rate of change (−0.106 [0.217] points per year; *P* = 0.627) between the two groups (Table 2 and Figure 2).

## 4 Discussion

The current study represents a pioneering effort to assess disease progression in PD patients with *BST1* rs4698412 variants through a longitudinal follow-up analysis. Our investigation reveals a more pronounced motor progression in PD patients carrying the *BST1* rs4698412 GA/AA genotype compared to those with the GG genotype. Specifically, our analysis indicates that the estimated rate of change in UPDRS-III score is 2.091 points per year higher in PD patients with the *BST1* rs4698412 GA/AA genotype than in those with the GG genotype. Furthermore, when comparing differences in motor domain progression rates, we observed that this greater progression in GA/AA carriers encompassed a more substantial increase in rigidity and bradykinesia score over time. Regarding cognitive progression, our analysis unveiled a similar estimated rate of change in MMSE score per year between the two groups.

Previous research has indicated a genetic correlation between *BST1* polymorphism rs4698412 and the predisposition to PD (Chang et al., 2011; Chang et al., 2015; Saad et al., 2011; Satake et al., 2009; Sharma et al., 2012; UK Parkinson’s Disease Consortium et al., 2011). While our study reveals that PD patients harboring the allelic variant A of *BST1* rs4698412 exhibit a more pronounced motor deterioration compared to GG homozygotes, the underlying mechanism by which these genotypes influence motor function remains inadequately elucidated. BST1, also referred to as CD157, belongs to the NADase/ADP-ribosyl cyclase family (Ferrero and Malavasi, 1997). Previous research suggests that BST1 may play a role in the molecular pathways involving cADPR formation and Ca^2+^ mobilization, acting as a neuro-regulator (Higashida et al., 2017). It has been postulated that an imbalance in calcium homeostasis within dopaminergic neurons could contribute to their degeneration and increase susceptibility to PD (Surmeier et al., 2010; Surmeier, 2007). Additionally, calcium signaling dynamics are integral in regulating diverse neuronal activities, encompassing the release of neurotransmitters and neuropeptides at inter-synaptic sites (Berridge et al., 2003; Soden et al., 2023). Furthermore, BST1 was initially identified as a surface receptor on Bone Marrow Stromal Cells (BMSCs) that stimulates the proliferation of pre-B cells (Kaisho et al., 1994). Recent studies have uncovered that as individuals age, BMSCs develop a senescence-associated secretory phenotype, releasing inflammatory cytokines such as IL-6, IL-8, IFN-γ, MCP-1/2, and TIMP-2, (Borgoni et al., 2021; Gonzalez-Meljem et al., 2018) and subsequently differentiating into age-associated B cells (ABCs) (Long et al., 2023). ABCs infiltrate the brain parenchyma and initiate the activation of microglia, subsequently giving rise to a state of sustained chronic inflammation (Wang et al., 2021). Therefore, we posit that the malfunction of BST1 could lead to hindered normal growth of pre-B cells, along with the plausible involvement of age-related BMSCs in triggering neuroinflammatory responses and disruptions in microenvironmental homeostasis. These combined factors may potentially contribute to the underlying pathological mechanisms of PD. Besides, experiments demonstrated that compared with wild-type mice, *BST1* knockout (*BST1*^–/–^) mice exhibited anxiety-related symptoms, depression-like behaviors, and impaired social interaction similar to those observed in PD patients, suggesting a potential role of BST1 in pre-motor symptoms of PD (Kasai et al., 2017; Lopatina et al., 2014).

Li et al. (2019) explored the *BST1* rs4698412 variant-brain function-behavior relationships by examining the Amplitude of low-frequency fluctuations (ALFF) signals of functional magnetic resonance imaging (fMRI) in PD patients. Their results showed that significantly decreased ALFF values in the right lingual gyrus and the ALFF values were negatively associated with TUG test time (*r* = −0.797) and postural and gait disturbance scores (*r* = −0.937) in *BST1* rs4698412 GA/AA carriers compared with GG carriers. This objective imaging evidence could, to some extent, help to explain the influence of allele A of *BST1* rs4698412 on a pathological process contributing to more severe motor symptoms during PD progression.

According to the existing literature, multiple studies have focused on the correlation between the progression of PD and genotypes. Individuals with Parkinson’s disease who carry distinct gene variants, such as *LRRK2* risk variants (G2385R, and/or R1628P, and/or S1647T) (Oosterveld et al., 2015), *SNCA* rs1045722/T (Luo et al., 2019), Parkin-related mutations (Sun et al., 2021), *GBA* (Winder-Rhodes et al., 2013), or *LRRK2* G2019S mutation (Oosterveld et al., 2015), have presented a diverse spectrum of disease progression patterns. Hence, considering genetic variability becomes imperative for gaining deeper insights into the underlying causes and mechanisms of the disease. The more substantial progression estimates observed in our study could offer valuable insights for the design of clinical trials involving emerging BST1-targeted agents. Furthermore, in our present study, we employed linear mixed-effects models for analyzing repeated measurements, a methodology capable of handling data imbalances arising from variations in the timing of the initial visit, unequal quantities of follow-up visits, and differing intervals between visits. This robust approach enhances the significance and value of our research findings.

Our study possesses several limitations that warrant consideration. Firstly, we exclusively examined the impact of the *BST-1* rs4698412 mutation on disease progression in PD patients, disregarding potential influences from other genetic variants, intricate gene-gene interactions, and the combined effects of gene-environment interactions. Secondly, while UPDRS remains the standard tools for assessing motor symptoms in PD, its inability to differentiate PD-specific progression from age-related functional decline must be acknowledged. Age-associated motor deficits (e.g., gait slowing, postural instability) may confound longitudinal assessments. Although we adjusted for major clinical variables (including comorbidities and lifestyle factors), unmeasured factors such as medication adherence, physical activity levels, and subclinical cerebrovascular disease could influence progression rates. This limitation underscores the necessity of integrating multidimensional biomarkers (e.g., cerebrospinal fluid profiles, blood-based biomarkers, neuroimaging metrics, and digital health parameters) with conventional clinical evaluations in future studies. Furthermore, the exclusive use of MMSE for cognitive evaluation may obscure domain-specific cognitive decline patterns in PD. The absence of a comprehensive neuropsychological assessment battery significantly limits our ability to characterize nuanced cognitive trajectories. Future investigations should incorporate detailed neuropsychological evaluations to better delineate cognitive progression patterns. Besides, the follow-up period in our study was relatively brief, and the number of follow-up visits was limited. The estimated progression rates should be interpreted with caution due to the moderate follow-up duration and variability in assessment intervals. Extended observation periods and standardized visit schedules would improve the accuracy of longitudinal trajectory modeling. Lastly, the restriction of our cohort to a southern Chinese Han population is indeed a limitation for generalizability. Future studies should consider multi-center collaborations or include diverse populations to validate these results across different ethnic and regional groups.

## 5 Conclusion

This present study provides novel insights into the disease progression of PD patients harboring *BST1* rs4698412 variants. Our findings indicated that individuals with PD who carry the *BST1* rs4698412 A-allelic variant exhibit more pronounced deterioration in motor function, as reflected by higher UPDRS-III score. Further research is warranted to unravel the underlying mechanisms driving these genotype-specific effects and to explore potential implications for personalized therapeutic interventions.

## Data Availability

The data analyzed in this study is subject to the following licenses/restrictions: the data are not publicly available due to privacy or ethical restrictions. Requests to access these datasets should be directed to the corresponding author, unionqyye8@fjmu.edu.cn.

## Supplementary material

Supplementary Figure 1 Flow chart of screening in this study.

Supplementary Figure 2 Spaghetti plots of changes in UPDRS-III scores over visit time.

Supplementary Figure 3 Spaghetti plots of changes in MMSE scores over visit time.
