# Supplementary material for: The impact of BST1 rs4698412 variant on Parkinson’s disease progression in a longitudinal study
Source: Front Aging Neurosci. 2025 Apr 16;17:1570347. doi: 10.3389/fnagi.2025.1570347 (PMC12040838; doi:10.3389/fnagi.2025.1570347)
Supplement: Supplementary file 1 [file Table_1.docx]

**Supplementary Table 1. Genotype frequencies for *BST1* rs4698412 in PD groups**

| Genotypes | Actual number of PD | Genotype frequency(%) | Theoretical umber of PD |
| --- | --- | --- | --- |
| GG | 74 | 40.6 | 74 |
| GA | 84 | 46.2 | 84 |
| AA | 24 | 13.2 | 24 |

Abbreviations: PD, Parkinson’s disease; BST1, bone marrow stromal cell antigen-1.

Genotype frequencies for *BST1* rs4698412 did not deviate from HWE in PD group (χ^2^ = 0, *P* = 1).
