# Supplementary material for: The impact of BST1 rs4698412 variant on Parkinson’s disease progression in a longitudinal study
Source: Front Aging Neurosci. 2025 Apr 16;17:1570347. doi: 10.3389/fnagi.2025.1570347 (PMC12040838; doi:10.3389/fnagi.2025.1570347)
Supplement: Supplementary file 2 [file Table_2.docx]

**Supplementary Table 2. Demographic and clinical characteristics of MMSE subjects**

|  | GG carriers | GA/AA carriers | *P* value |
| --- | --- | --- | --- |
| Patients, n | 73 | 104 | NA |
| Male, n (%) | 41 (56.2) | 53 (51.0) | 0.408^a^ |
| Age at baseline, Y | 61.0 (54.0, 69.0) | 63.0 (54.3, 69.0) | 0.302^b^ |
| Age at onset, Y | 56.82 ± 9.67 | 58.06 ± 10.58 | 0.429^c^ |
| Disease duration at baseline, y | 3.0 (2.0, 6.0) | 3.0 (1.0, 5.0) | 0.241^b^ |
| Education, y | 9.0 (6.0, 12.0) | 9.0 (6.0, 12.0) | 0.428^b^ |
| LEDD at baseline | 375.0 (300.0, 450.0) | 325.0 (300.0, 437.5) | 0.279^b^ |
| UPDRS-III score at baseline | 23.0 (16.5, 30.0) | 22.5 (17.0, 33.0) | 0.876^b^ |
| MMSE score at baseline | 27.0 (24.0, 28.0) | 26.0 (23.0, 28.0) | 0.640^b^ |
| Hypertension, n (%) | 16 (21.9) | 21 (20.2) | 0.781^a^ |
| Diabetes, n (%) | 5 (6.8) | 7 (6.7) | 0.975^a^ |
| Smoking, n (%) | 7 (9.6) | 9 (8.7) | 0.831^a^ |
| Drinking, n (%) | 4 (5.5) | 3 (2.983) | 0.383^a^ |

Abbreviations: UPDRS, Unified Parkinson’s Disease Rating Scale; y, Years; Y, Years old; MMSE, Mini-Mental State Examination; LEDD, levodopa equivalent daily dose; NA, not applicable.

Variables with normal distribution were represented as mean ± standard deviation, while variables with skewed distribution were expressed as median and interquartile range.

^a^ Chi-square Test

^b^ Mann-Whitney U Test

^c^ Two-independent samples t-test
