# Supplementary material for: The impact of BST1 rs4698412 variant on Parkinson’s disease progression in a longitudinal study
Source: Front Aging Neurosci. 2025 Apr 16;17:1570347. doi: 10.3389/fnagi.2025.1570347 (PMC12040838; doi:10.3389/fnagi.2025.1570347)
Supplement: Supplementary file 3 [file Table_3.docx]

**Supplementary Table 3. Cross-Sectional Study of *BST1* rs4698412 GG carriers and GA/AA carriers**

| **The impact of genotypes on motor function (UPDRS-III scores) at baseline** | | | |
| --- | --- | --- | --- |
|  | Coefficient estimate | Standard error | *P* value |
| Genotype ^a^ | 1.413 | 1.483 | 0.342 |
| Gender ^b^ | -2.036 | 1.631 | 0.214 |
| Age at baseline | 0.051 | 0.081 | 0.529 |
| Disease duration at baseline | 0.807 | 0.208 | **0.000** |
| Years of Education | -0.402 | 0.197 | **0.043** |
| MMSE score at baseline | -0.466 | 0.240 | 0.054 |
| LEDD at baseline | 0.002 | 0.004 | 0.629 |
| Hypertension ^c^ | -0.947 | 1.924 | 0.623 |
| Diabetes ^c^ | 1.829 | 2.998 | 0.543 |
| Smoking ^c^ | 1.521 | 2.731 | 0.578 |
| Drinking ^c^ | -3.938 | 3.953 | 0.321 |
|  |  |  |  |
| **The impact of genotypes on cognitive function (MMSE scores) at baseline** | | | |
|  | Coefficient estimate | Standard error | *P* value |
| Genotype ^a^ | -0.371 | 0.449 | 0.411 |
| Gender ^b^ | -0.139 | 0.497 | 0.780 |
| Age at baseline | -0.050 | 0.024 | **0.039** |
| Disease duration at baseline | -0.081 | 0.066 | 0.219 |
| Years of Education | 0.419 | 0.050 | **0.000** |
| UPDRS-III score at baseline | -0.044 | 0.023 | 0.054 |
| LEDD at baseline | 0.002 | 0.001 | 0.107 |
| Hypertension ^c^ | 0.674 | 0.586 | 0.252 |
| Diabetes ^c^ | -1.217 | 0.898 | 0.177 |
| Smoking ^c^ | 0.127 | 0.820 | 0.877 |
| Drinking ^c^ | -0.813 | 1.190 | 0.495 |

Abbreviations: UPDRS, Unified Parkinson’s Disease Rating Scale; MMSE, Mini-Mental State Examination; LEDD, levodopa equivalent daily dose. Bold values indicate statistically significant differences at *p* < 0.05.

^a^ *BST1* rs4698412 GA/AA carriers vs. GG carriers

^b^ Female vs. male

^c^ Yes vs. no
