# Supplementary figures and images for: The impact of BST1 rs4698412 variant on Parkinson’s disease progression in a longitudinal study
Source: Front Aging Neurosci. 2025 Apr 16;17:1570347. doi: 10.3389/fnagi.2025.1570347 (PMC12040838; doi:10.3389/fnagi.2025.1570347)

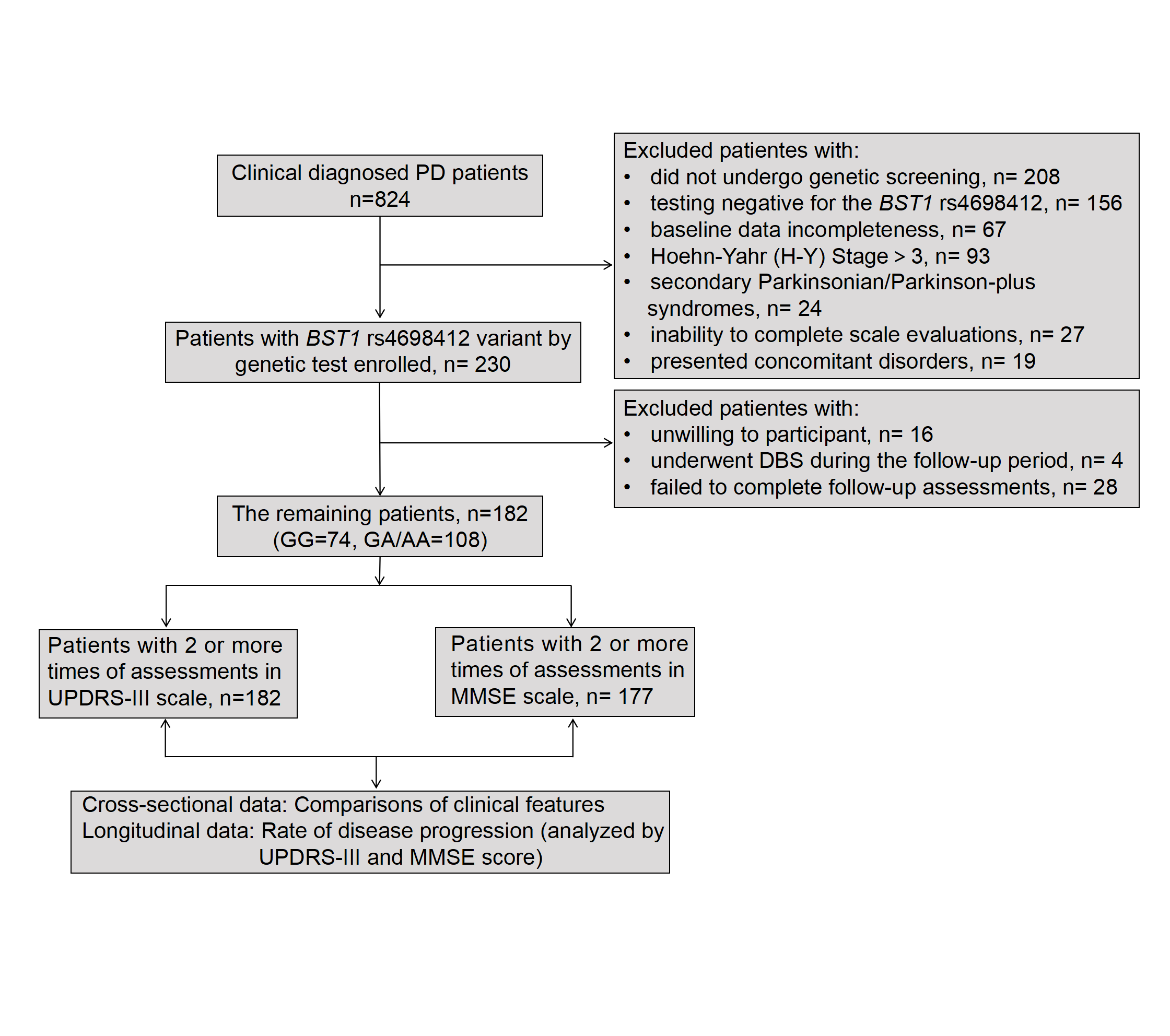

Supplement: Supplementary Figure 1 — Flow chart of screening in this study. [file Image_1.tif]

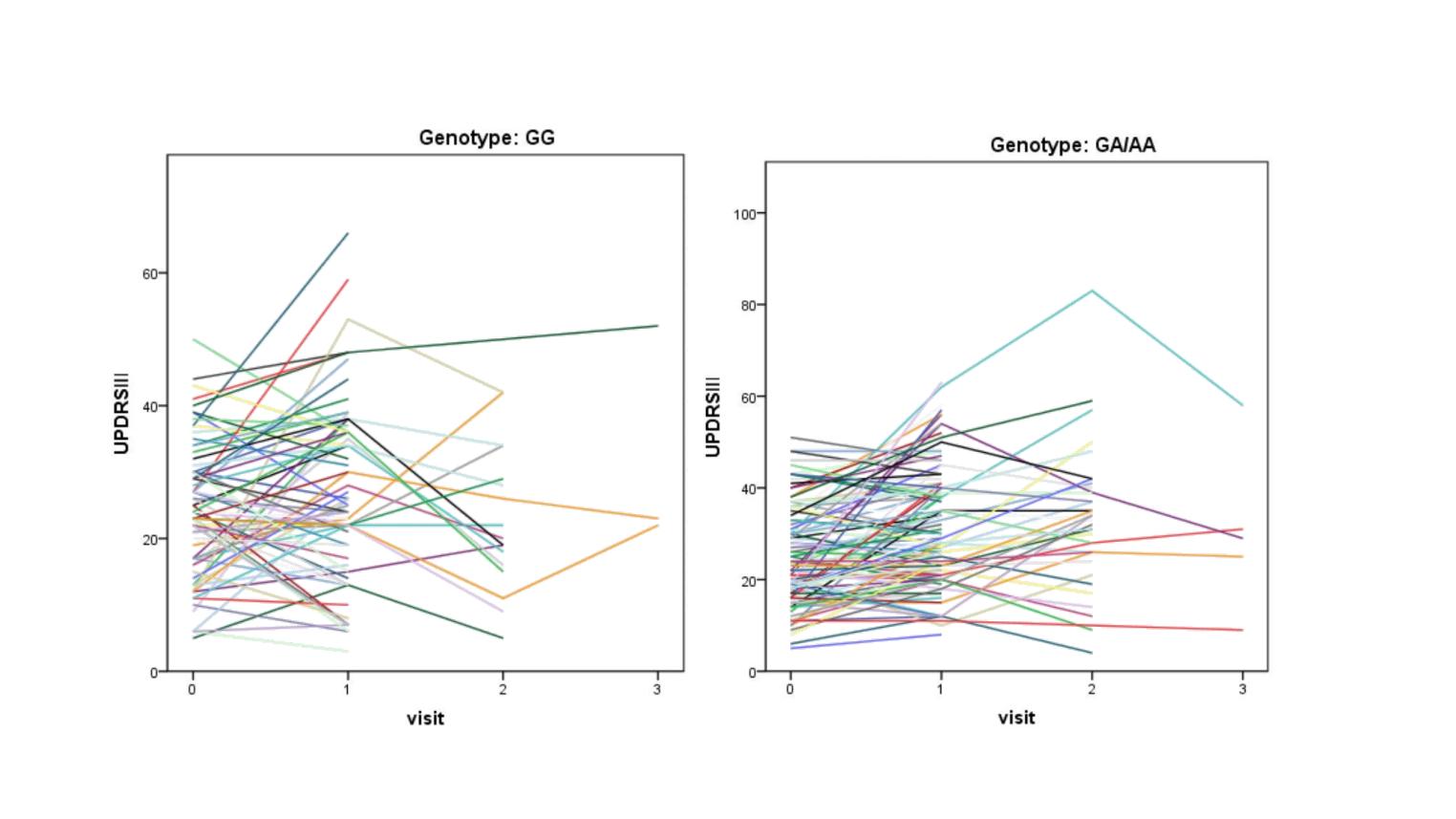

Supplement: Supplementary Figure 2 — Spaghetti plots of changes in UPDRS-III scores over visit time. [file Image_2.jpeg]

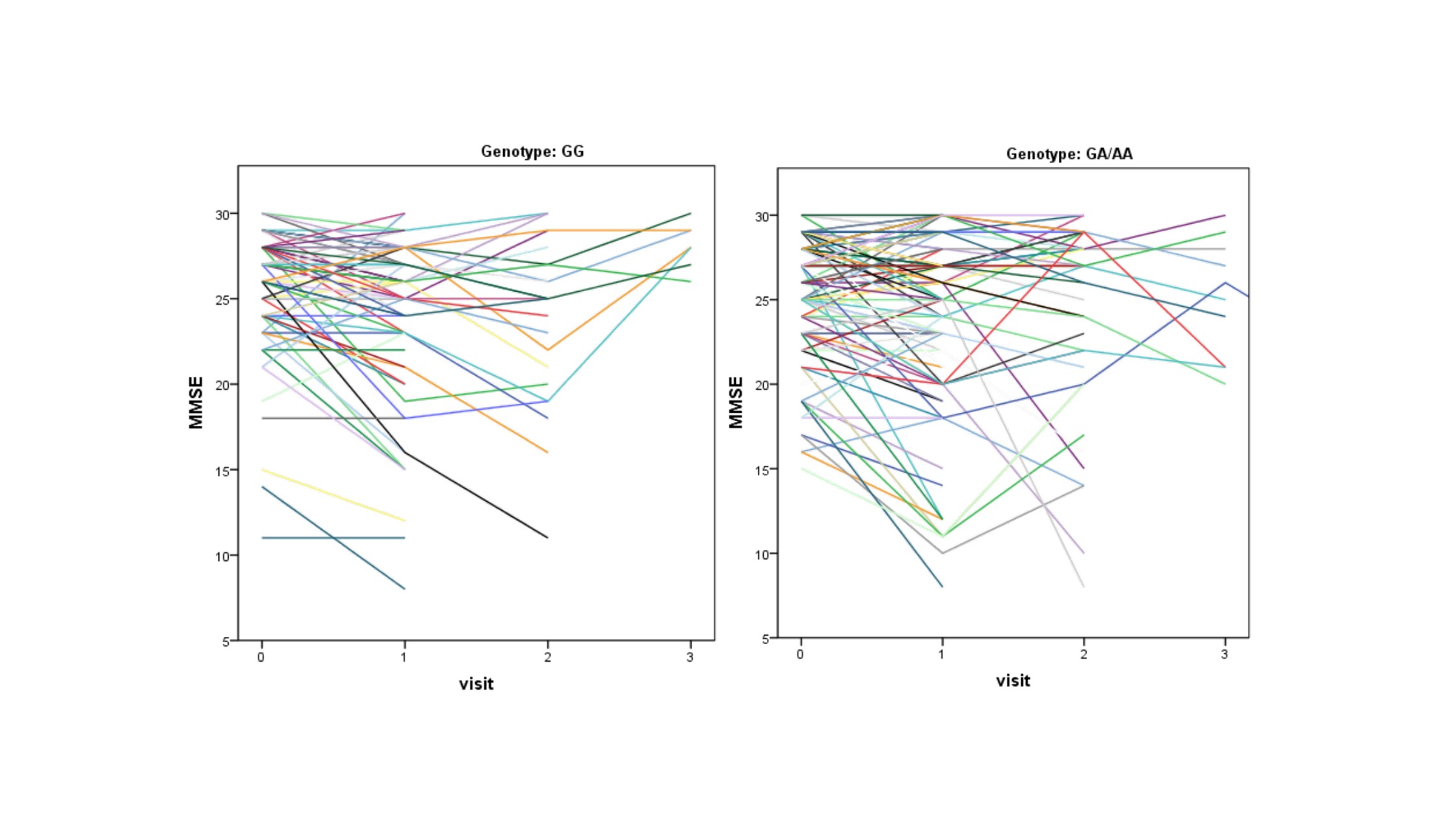

Supplement: Supplementary Figure 3 — Spaghetti plots of changes in MMSE scores over visit time. [file Image_3.jpeg]
